# Substance Use Disorders in Elderly Admissions to an Academic Psychiatric Inpatient Service over a 10-Year Period

**DOI:** 10.1155/2016/4973018

**Published:** 2016-10-19

**Authors:** Dennis Dombrowski, Nelly Norrell, Suzanne Holroyd

**Affiliations:** ^1^Department of Psychiatry and Neurobehavioral Sciences, University of Virginia, Charlottesville, VA, USA; ^2^Department of Psychiatry and Behavioral Medicine, Marshall University, Huntington, WV, USA

## Abstract

*Objective*. There is a paucity of research on substance use disorders (SUDs) in the elderly psychiatric population. This study examines SUDs in a geriatric psychiatry inpatient service over a 10-year period.* Methods*. Data from 1788 elderly psychiatric inpatients from a ten-year period was collected. Variables collected included psychiatric diagnoses, SUD, number of psychiatric admissions, and length of stay. Those with and without a SUD were compared using Chi-Square or Student's *t*-test as appropriate using SPSS.* Results*. 11.7% (*N* = 210) of patients had a SUD, and the most common substance was alcohol at 73.3% (*N* = 154) or 8.6% of all admissions. Other SUDs were sedative-hypnotics (11%), opiate (2.9%), cannabis (1%), tobacco (1.4%), and unspecified SUD (38.6%). SUD patients were significantly younger, divorced, male, and less frequently readmitted and had shorter lengths of stay. The most common comorbid diagnoses were major depression (26.1%), bipolar disorder (10.5%), and dementia (17.1%).* Conclusions*. Over 10% of psychogeriatric admissions were associated with a SUD, with alcohol being the most common. Considering the difficulties in diagnosing SUD in this population and the retrospective study design, the true prevalence in elderly psychiatric inpatients is likely higher. This study adds to sparse literature on SUD in elderly psychiatric patients.

## 1. Introduction

Substance use disorders (SUD) are common in the geriatric population, and multiple studies have found negative health outcomes associated with SUD in this population [1–4]. Elderly patients with SUDs have been found to be at greater risk of falls and delirium [1, 2] and to have negative effects on comorbid diagnoses such as hypertension, diabetes, gastritis, anemia, and dementia [3]. Alcohol use has also been linked to suicide in elderly patients and appears to be an independent predictor of suicide risk in both sexes [4].

However, surprisingly little research has been done on SUDs in the geriatric psychiatric population. While prevalence studies of alcohol use disorders in the United States have been done in various geriatric medical populations showing rates of alcohol use disorders ranging from 14 to 21% [5, 6], there are few in the psychiatric geriatric population. One study examining geriatric psychiatry outpatients found a 20% prevalence of SUD, mainly alcohol, narcotics, and benzodiazepines [7]. One examining psychiatric older inpatients (ages 55 and up) over a 2-year period revealed a 21% prevalence of SUD, most commonly alcohol, that was associated with mood and personality disorders [8]. Another found a prevalence of alcohol use disorders of 23–44% among psychiatric inpatients [3]. Since the 1990s, there is evidence that illegal substance use is increasing in older adults [9–11], yet there has been no further study of SUDs in the geriatric psychiatric population.

SUD diagnoses are often missed by the clinicians treating the elderly due to a variety of factors including provider attitudes that elderly do not have SUDs and thus they have a low degree of suspicion when assessing the elderly [3, 5, 12]. Increased data regarding the prevalence of SUDs could raise awareness of the problem and hence better the efforts on the part of clinicians in diagnosing such disorders.

In this study, the prevalence of SUDs in geriatric psychiatry inpatients over a 10-year period was determined as well as examining associated clinical and demographic characteristics between elderly psychiatric patients with and without SUDs.

## 2. Methods

Information over a 10-year period (1999–2009) was collected from an electronic database which logs the clinical and demographic information of all patients admitted to a psychiatric inpatient unit at an academic center. There were 1,788 admissions for patients 65 and over during this time period and all were included in this study. Diagnoses were made using DSM IV criteria. Data was deidentified and analyzed using SPSS. Patients were grouped based on the presence or absence of a SUD, which included intoxication, withdrawal, abuse, dependence, and substance induced disorders. These groups were then compared on clinical and demographic variables including age and length of stay, using *t*-test analysis, and gender, race, marital status, and history of multiple admissions using Chi-Square and Fisher's exact test analysis. *p* value ≤ 0.05 was used to denote significance. The Institutional Review Board reviewed and approved this study.

## 3. Results

The mean age for these patients was found to be 75.5 years ± 7.7 years. The frequency of geriatric admissions per year during this time period ranged from 139 to 202. 1082 (60.5%) were female and 702 (39.3%) were male. 1455 (81.4%) were white while 276 (15.4%) were black, 2 (0.1%) were Asian, 2 (0.1%) were Hispanic, and 44 (2.5%) were of “other” ethnic backgrounds. Marital status was noted as 671 (37.5%) married, 552 (30.9%) widowed, 277 (15.5%) single, and 229 (12.8%) divorced. The average length of stay was 13.9 days ± 14.2 days.

Of all 1,788 admissions, 11.7% (*N* = 210) were associated with at least one SUD. Within this group of 210 patients, the most commonly used substance was alcohol with associated disorders totaling 73.3% (*N* = 154) which included the following diagnoses: alcohol intoxication (*N* = 9), alcohol abuse (*N* = 31), alcohol dependence (*N* = 78), alcohol withdrawal (*N* = 16), alcohol induced dementia (*N* = 14), and alcohol induced mental disorder (*N* = 6). The prevalence of other SUDs was as follows: sedative-hypnotic abuse/dependence 11% (*N* = 23), opiate abuse/dependence 2.9% (*N* = 6), cannabis abuse 1% (*N* = 2), tobacco use disorder 1.4% (*N* = 3), and unspecified drug induced disorders/withdrawal 38.6% (*N* = 81). The diagnoses associated with unspecified drugs included the following: drug induced delirium (*N* = 16), drug induced mood disorder (*N* = 16), drug induced psychosis (*N* = 4), drug abuse (*N* = 3), drug induced mental disorder (*N* = 5), and drug withdrawal (*N* = 37).

Next, patients with a SUD were compared to those without a SUD (Table 1). SUD patients were significantly younger and had shorter lengths of stay in the hospital. Men were more likely to have SUDs compared to women. The proportion of divorced patients was higher in those with a SUD as compared to other marital statuses. Interestingly, patients with a SUD were also less likely to have readmissions to the psychiatric unit compared to those without SUD. There was no difference in race or other psychiatric diagnoses between those with or without a SUD. There was no difference in SUD prevalence over the 10-year period.

The most frequently comorbid Axis I diagnoses seen in patients with a SUD were major depression 26.1% (*N* = 55), bipolar affective disorder type I 10.5% (*N* = 22), vascular dementia 10% (*N* = 21), Alzheimer's type dementia 7.1% (*N* = 15), adjustment disorder 7.1% (*N* = 15), delirium 4.8% (*N* = 10), and generalized anxiety disorder 2.9% (*N* = 6).

## 4. Discussion

The prevalence of SUD in a geriatric inpatient population admitted over a ten-year period was 11.7%. Not surprisingly, the most commonly abused substance was found to be alcohol totaling 73.3% of the identified substance use disorders, in line with previous studies. However, other SUDs were also found including sedative-hypnotics, opiates, cannabis, and tobacco. The relatively higher age of our population may explain why other SUDs such as cannabis were not seen at higher rates. It is unknown whether males truly have higher rates of SUD or whether they are more likely to reveal their SUD use as compared to females or if clinicians have a higher rate of suspicion in males; however, substance use disorders are more common in males over the life span, so our data is consistent with that finding.

Identification of alcohol as the primary drug of abuse in elderly populations is consistent with previous research which has also linked alcohol use to depression, suicide, falls, and increased medical morbidity. In this study, SUDs were found to be most frequently comorbid with affective disorders, primarily major depression and bipolar affective disorder type I. But a range of other diagnoses including dementia, adjustment disorder, delirium, and generalized anxiety disorder were also found to be associated with substance use. As such, evaluation and treatment for mood disturbances, confusion, or anxiety in this population should also include a thorough assessment for underlying SUD.

The major limitation of this study is that it is retrospective. Thus, the prevalence of SUD in this population is likely greater than that identified. Another limitation is that some patients were readmitted over the 10-year period and were counted more than once. Thus this study is a prevalence of SUD in psychiatric admissions over a 10-year period rather than the prevalence among psychiatric patients over a 10-year period. Despite these limitations, this study is the first to evaluate the prevalence of SUD in a geriatric psychiatric inpatient population over an extended period and in such a large sample size. Since more than one in ten admissions were found to be associated with a SUD, this emphasizes the importance of assessing elderly psychiatric patients for the presence of such disorders which might otherwise present covertly such as falls, cognitive impairment, or depression. Given the paucity of data on SUD in the geriatric psychiatric population, further study is clearly warranted.

## Figures and Tables

**Table 1:** Comparison of elderly psychiatric inpatients with and without a SUD.

	SUD (*N* = 210)	NO SUD (*N* = 1578)	Significance
Age (years)	74.3 ± 8.7	75.6 ± 7.6	*p* = 0.02 (*t*-test)

Length of stay (days)	10.7 ± 9.4	14.3 ± 14.6	*p* < 0.001 (*t*-test)

Gender:	*N* (%)	*N* (%)	
Men	110 (52.4%)	592 (37.5%)	*p* < 0.001 (Pearson Chi-Square, 2-sided)
Women	100 (47.6%)	982 (62.2%)	

Marital status:	*N* (%)	*N* (%)	
Married	78 (37.1%)	593 (37.5%)	*p* = 0.02 (Pearson Chi-Square, 2-sided)
Divorced	41 (19.5%)	188 (11.9%)	
Single	34 (16.2%)	243 (15.4%)	
Widowed	50 (23.8%)	502 (31.8%)	
Separated	7 (3.3%)	52 (3.2%)	

Multiple psychiatric admissions	*N* (%)	*N* (%)	
Yes	80 (38.1%)	788 (49.9%)	*p* = 0.002 (Fisher exact test)
No	130 (61.9%)	790 (50.0%)	

## References

[1] Finkelstein E., Prabhu M., Chen H. (2007). Increased prevalence of falls among elderly individuals with mental health and substance abuse conditions. *American Journal of Geriatric Psychiatry*.

[2] Onen S.-H., Onen F., Mangeon J.-P., Abidi H., Courpron P., Schmidt J. (2005). Alcohol abuse and dependence in elderly emergency department patients. *Archives of Gerontology and Geriatrics*.

[3] Goldstein M. Z., Pataki A., Webb M. T. (1996). Alcoholism among elderly persons. *Psychiatric Services*.

[4] Waern M. (2003). Alcohol dependence and misuse in elderly suicides. *Alcohol and Alcoholism*.

[5] O'Connell H., Chin A.-V., Cunningham C., Lawlor B. (2003). Alcohol use disorders in elderly people—redefining an age old problem in old age. *British Medical Journal*.

[6] Curtis J. R., Geller G., Stokes E. J., Levine D. M., Moore R. D. (1989). Characteristics, diagnosis, and treatment of alcoholism in elderly patients. *Journal of the American Geriatrics Society*.

[7] Holroyd S., Duryee J. J. (1997). Substance use disorders in a geriatric psychiatry outpatient clinic: prevalence and epidemiologic characteristics. *Journal of Nervous & Mental Disease*.

[8] Speer D. C., Bates K. (1992). Comorbid mental and substance disorders among older psychiatric patients. *Journal of the American Geriatrics Society*.

[9] Patterson T. L., Jeste D. V. (1999). The potential impact of the baby-boom generation on substance abuse among elderly persons. *Psychiatric Services*.

[10] Han B., Gfroerer J. C., Colliver J. D., Penne M. A. (2009). Substance use disorder among older adults in the United States in 2020. *Addiction*.

[11] Simoni-Wastila L., Yang H. K. (2006). Psychoactive drug abuse in older adults. *The American Journal Geriatric Pharmacotherapy*.

[12] McInnes E., Powell J. (1994). Drug and alcohol referrals: are elderly substance abuse diagnoses and referrals being missed?. *British Medical Journal*.

